# Exclusively ocular and cardiac manifestation of granulomatosis with polyangiitis – a case report

**DOI:** 10.1186/s12886-019-1148-4

**Published:** 2019-06-28

**Authors:** Małgorzata Rogaczewska, Mariusz Puszczewicz, Marcin Stopa

**Affiliations:** 1 Department of Ophthalmology, Chair of Ophthalmology and Optometry, Poznan University of Medical Sciences, ul. Grunwaldzka 16/18, 60-780 Poznan, Poland; 2 Department of Rheumatology and Internal Diseases, Poznan University of Medical Sciences, ul. 28 Czerwca 1956 r. 135/147, 61-545 Poznan, Poland

**Keywords:** Granulomatosis with polyangiitis, Conjunctivitis, Panuveitis, Retinal detachment, Heart block

## Abstract

**Background:**

Granulomatosis with polyangiitis (GPA) is an antineutrophil cytoplasmic antibodies (ANCA)-associated necrotizing granulomatous vasculitis that affects small to medium size vessels. While the classical form with renal and respiratory tract involvement is mainly seen, a limited form (i.e., with no renal disease) may also occur. We present an unusual case of GPA manifesting merely as a bilateral ocular involvement and complete heart block.

**Case presentation:**

We report a case of a 60-year-old male patient with a limited form of GPA who initially presented with bilateral chronic conjunctivitis and complete atrioventricular block. His visual acuity subsequently declined due to progression to bilateral panuveitis with exudative retinal detachment. The laboratory investigation revealed the elevation of acute phase reactants and strongly positive cytoplasmic ANCA (c-ANCA). Despite negative conjunctival and musculocutaneous biopsy results, the positive c-ANCA, and the clinical manifestation, i.e., heart and ocular involvement, led to the diagnosis of GPA. The remission was achieved with cyclophosphamide and methylprednisolone systemic therapy.

**Conclusions:**

A limited form of GPA may be a diagnostic chameleon. Though rare, it is essential to consider even extremely uncommon findings. Our patient is the first case of such a unique demonstration of the limited GPA manifesting as a bilateral ocular involvement and complete heart block.

## Background

Granulomatosis with polyangiitis (GPA) is a granulomatous systemic vasculitis involving small to medium-sized arteries and veins. While the kidneys and respiratory tract are predominantly affected, a limited form of GPA may disclose as a single organ disease [1, 2]. Here, we report a case of granulomatosis with polyangiitis manifesting merely as a bilateral ocular involvement and complete heart block. To our knowledge, this is the first case of such an unusual presentation of GPA.

## Case presentation

A 60-year-old Caucasian male presented to the Department of Ophthalmology in Poznan with a complaint of bilateral ocular redness, pain, severe photophobia, and progressive deterioration of vision in April 2015.

Three months earlier, he had been diagnosed with bilateral conjunctivitis, which did not respond to standard treatment. His past medical history was significant for hypertension and tinnitus of the right ear for several months. No other symptoms or signs of systemic diseases were recorded.

In the meantime, the patient was admitted to the Department of Cardiology-Intensive Therapy with cardiogenic shock due to complete atrioventricular (AV) block. He underwent temporary pacing, followed by permanent dual-chamber pacemaker insertion. Two weeks later, because of the exacerbation of his eyes problems, he was referred to us with the diagnosis of bilateral anterior uveitis.

At presentation, his best-corrected visual acuity (BCVA) in the right eye (RE) was 0.7 and in the left eye (LE) was 0.25. The corneal reflex of the LE was decreased.

**Fig. 1:**
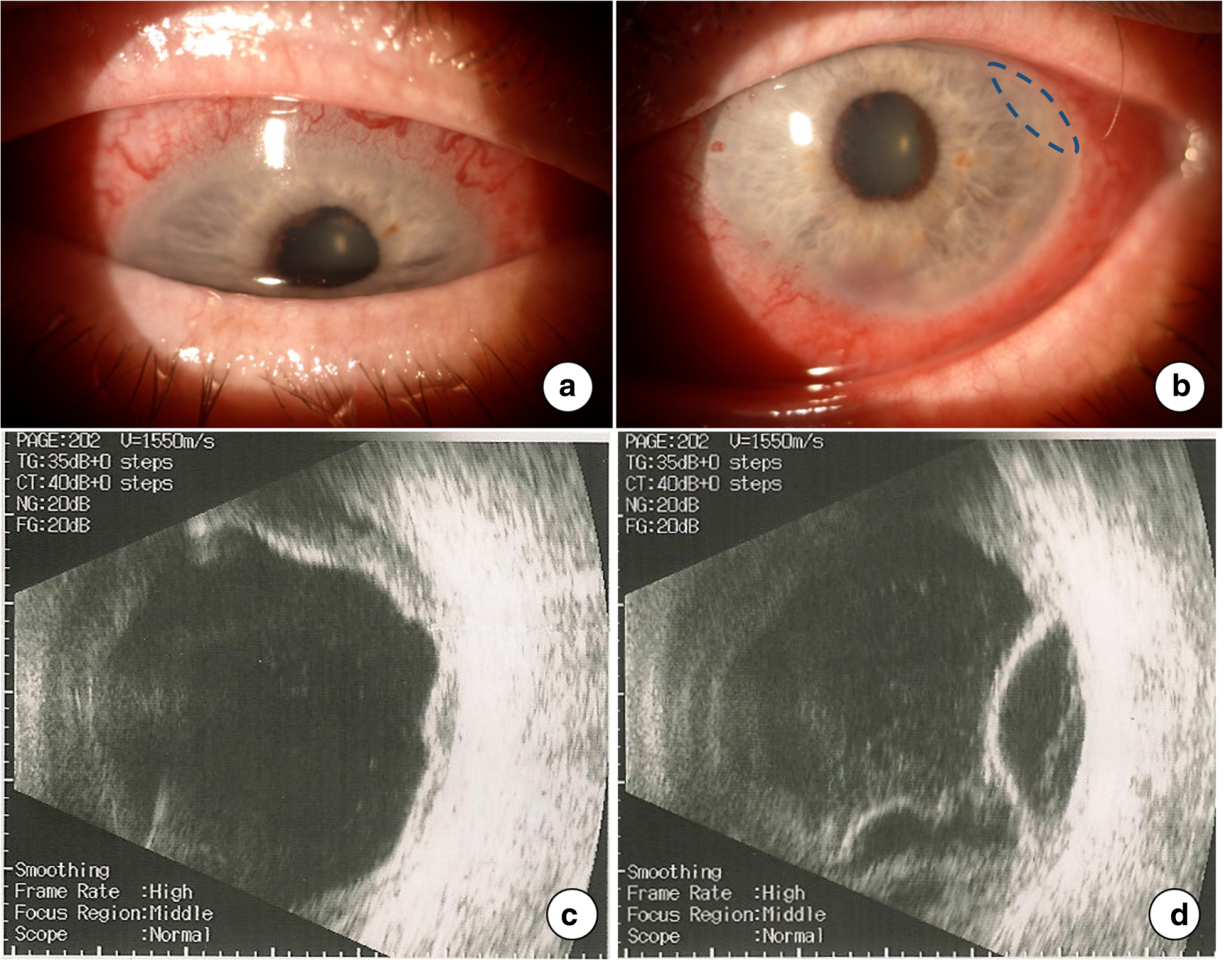
Examination at presentation. Diffuse scleritis, mild paralimbal keratitis and posterior synechiae in the right (**a**) and left eye (**b**). Ultrasound examination showed inflammation of the vitreous body and exudative retinal detachment in the right (**c**) and left eye (**d**). The blue dashed line delineates the location of keratitis

**Fig. 2:**
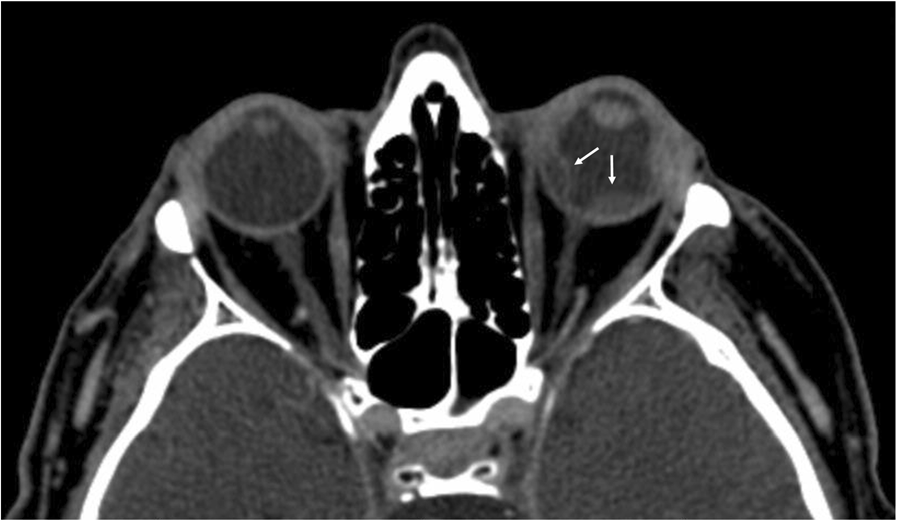
Computed tomography of the orbits. Bilateral anterior scleritis, and retinal detachment in the left eye (arrows)

Ocular examination revealed a non-necrotizing diffuse scleritis, mild paralimbal keratitis, anterior chamber cells (1+) and flare (2+), and posterior synechiae in both eyes, more marked in the LE (Fig. 1, a and b). The view of the fundus with indirect ophthalmoscope was limited, and the quality of standard photographic documentation was inadequate. Ultrasound evaluation elicited bilateral inflammation of the vitreous body, and exudative retinal detachment (Fig. 1, c and d). Head computed tomography scans revealed anterior inflammation of the eyewall, retinal detachment, and an enlargement of the left lacrimal gland (Fig. 2).

Due to progressive visual acuity decline (0.25 in RE; hand motion in LE) within a week, accompanied by the elevation of acute phase reactants, the detailed diagnostic investigation was performed.

The erythrocyte sedimentation rate, C-reactive protein, and plasma fibrinogen levels were increased, reaching maximum levels of 88 mm/h, 67 mg/l, and 968 mg/dl, respectively. Serological test for toxocariasis, Lyme disease, tuberculosis, syphilis, viral hepatitis, HIV, rheumatoid factor, anti-CCP, and tumor markers were negative. Despite elevated IgG antibody titers of toxoplasmosis, HSV-1, and CMV, they were not of diagnostic importance. Strongly positive serum cytoplasmic ANCA (c-ANCA), which specifically react with proteinase 3, showed a diffuse granular cytoplasmic staining pattern in a method of indirect immunofluorescence. The urinalysis was unremarkable, and serum creatinine level (0.84 mg/dl), as well as estimated glomerular filtration rate (115.02 ml/min/1.73 m^2^), were within the normal range. A radiographic study showed a narrowing of right sacroiliac joint space and no chest abnormalities. Abdominal ultrasound examination was normal. Our patient was also HLA-B27 positive.

Because c-ANCA were highly specific for GPA, conjunctival and musculocutaneous biopsies were obtained. The histopathological examination did not disclose any evidence of the disease.

Notwithstanding the negative biopsy results, we made a tentative diagnosis of GPA based merely on positive c-ANCA and ocular involvement.

**Fig. 3:**
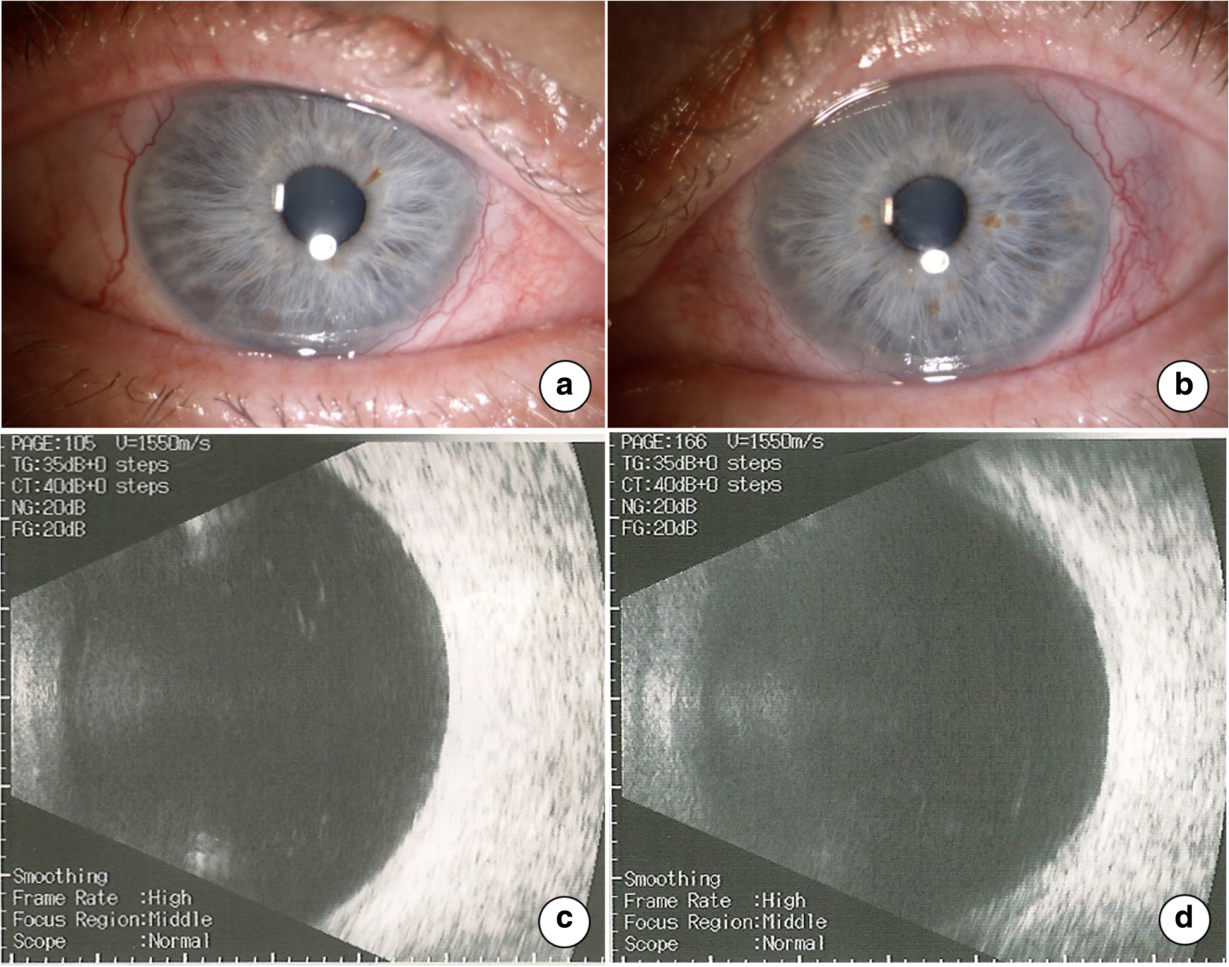
Examination after the second cycle of treatment with cyclophosphamide along with oral steroids. Scleral inflammation diminished (**a**, **b**) with complete resolution of vitritis and exudative retinal detachment in the right (**c**) and left eye (**d**)

The patient was referred to the Department of Rheumatology and Internal Medicine, where our diagnosis of GPA was upheld. The patient started therapy with cycles of intravenous steroids and cyclophosphamide along with oral steroids q.d. The response to the treatment was excellent, and ocular inflammation diminished. After the second cycle of therapy, his BCVA increased to 1.0 in RE and 0.2 in LE. The vitritis and exudative retinal detachment resolved completely (Fig. 3). Uneventful cataract surgery was performed in the LE that further enhanced his vision to 0.5 after 3 months. At this time, the total dose of cyclophosphamide administered over the 3 years was 9800 mg.

## Discussion

The most remarkable observation that emerges from our report is that granulomatosis with polyangiitis can manifest merely as a bilateral ocular involvement and complete heart block.

GPA is a systemic, ANCA-associated vasculitis involving small to medium-sized arteries and veins, accompanied by necrotizing granulomatous inflammation. Generally, this rare noninfectious entity manifests in a classical form, i.e., with respiratory tract and kidneys involvement. A limited form of GPA, encompassing only one or two organ systems (but with no renal disease), occurs significantly less often [1, 2]. Patients may also present with nonspecific signs and symptoms, i.e., fever, weight loss, and arthralgias. Thus the diagnosis could be made according to the American College of Rheumatology 1990 criteria for the classification of GPA [3].

Antineutrophil cytoplasmic antibodies play an essential role in the diagnostic process. The presence of c-ANCA, a highly specific hallmark of GPA, may lead to the definitive diagnosis when cogent clinical features are not seen. Additionally, a biopsy of the affected organ revealing necrotizing vasculitis or granulomatous inflammation depicts histological evidence of the disease [1].

The most typical clinical presentation of GPA comprises sinusitis, pulmonary infiltrates, and necrotizing glomerulonephritis. Although ocular involvement was found in even up to 60% of patients, as the initial manifestation of the disease, it was only present in 8–16% of cases. Patients may have a variety of ocular findings, including episcleritis, scleritis, peripheral ulcerative keratitis, and proptosis. However, conjunctivitis, uveitis, and lacrimal gland enlargement are rarely found [1, 2, 4–6]. Cardiac disorders are not commonly seen (3,3–10%) [1, 7]. Furthermore, AV block was described in several studies [8, 9], but only three cases with nasal/sinus involvement and AV block in the limited form of GPA were previously reported [10–12]. Thus, our patient is the first case of limited GPA manifesting as a bilateral ocular involvement and complete heart block.

The differential diagnosis of both ocular and cardiac involvement should include such entities as Lyme disease, sarcoidosis, and HLA B27-associated disorders [13–18]. On the other hand, separate ocular and cardiac disorders that coexist can manifest these signs and symptoms. Therefore, a detailed analysis of clinical features and laboratory findings plays a crucial role in establishing the final diagnosis.

Due to possible fulminant nature of the disease, which is a rare but life-threatening condition, the prompt detection and treatment are essential [8, 12]. Combination therapy with cyclophosphamide and corticosteroids is used to induce remission. If it is not effective, biologic treatment (rituximab) may be considered [1].

## Conclusions

Our findings demonstrated that conjunctivitis and heart block alone could be the only initial manifestation of GPA. Importantly, these symptoms may result from other unrelated diseases. Therefore the underlying etiology can be just overlooked. We are confident that our observation has clinical value for either ophthalmologists and rheumatologists.

## Data Availability

All the data supporting our findings is contained within the manuscript. More clinical data if necessary, is available from the corresponding author on request.

## Abbreviations

ANCA: Antineutrophil cytoplasmic antibodies
Anti-CCP: Anti-cyclic citrullinated peptide
AV: Atrioventricular
BCVA: Best-corrected visual acuity
c-ANCA: Cytoplasmic antineutrophil cytoplasmic antibodies
CMV: Cytomegalovirus
GPA: Granulomatosis with polyangiitis
HIV: Human immunodeficiency virus
HLA-B27: Human leukocyte antigen B27
HSV-1: Herpes simplex virus 1
LE: Left eye
RE: Right eye
